# Supplementary material for: Unveiling the Bioactive Potential of the Invasive Jellyfish Phyllorhiza punctata Through Integrative Transcriptomic and Proteomic Analyses
Source: Biomolecules. 2025 Aug 4;15(8):1121. doi: 10.3390/biom15081121 (PMC12383608; doi:10.3390/biom15081121)
Supplement: Supplementary file 1 [file biomolecules-15-01121-s001.zip › Suplementary Material/FileS3/PEPFOLD4/XP_065056972_14350/clusters.html]

 PEP-FOLD clusters


Download archive of all models  here   

|  |  |  |
| --- | --- | --- |
| # | file | sOPEP |
| # cluster 1 - Size: 13 |  |  |
|  | XP\_065056972\_14350-model1 | -10.4005 |
|  | XP\_065056972\_14350-model1.1 | -10.0462 |
|  | XP\_065056972\_14350-model1.2 | -10.0013 |
|  | XP\_065056972\_14350-model1.3 | -9.92767 |
|  | XP\_065056972\_14350-model1.4 | -9.88656 |
|  | XP\_065056972\_14350-model1.5 | -9.84108 |
|  | XP\_065056972\_14350-model1.6 | -9.7924 |
|  | XP\_065056972\_14350-model1.7 | -9.64404 |
|  | XP\_065056972\_14350-model1.8 | -9.63582 |
|  | XP\_065056972\_14350-model1.9 | -9.56275 |
|  | XP\_065056972\_14350-model1.10 | -9.56173 |
|  | XP\_065056972\_14350-model1.11 | -9.19857 |
|  | XP\_065056972\_14350-model1.12 | -8.41622 |
| # cluster 2 - Size: 23 |  |  |
|  | XP\_065056972\_14350-model2 | -10.2549 |
|  | XP\_065056972\_14350-model2.1 | -10.2549 |
|  | XP\_065056972\_14350-model2.2 | -10.2549 |
|  | XP\_065056972\_14350-model2.3 | -10.2549 |
|  | XP\_065056972\_14350-model2.4 | -9.52794 |
|  | XP\_065056972\_14350-model2.5 | -9.52794 |
|  | XP\_065056972\_14350-model2.6 | -9.4666 |
|  | XP\_065056972\_14350-model2.7 | -9.45005 |
|  | XP\_065056972\_14350-model2.8 | -9.45005 |
|  | XP\_065056972\_14350-model2.9 | -9.44175 |
|  | XP\_065056972\_14350-model2.10 | -9.41746 |
|  | XP\_065056972\_14350-model2.11 | -9.41746 |
|  | XP\_065056972\_14350-model2.12 | -9.40321 |
|  | XP\_065056972\_14350-model2.13 | -9.31771 |
|  | XP\_065056972\_14350-model2.14 | -9.31771 |
|  | XP\_065056972\_14350-model2.15 | -9.09881 |
|  | XP\_065056972\_14350-model2.16 | -9.08011 |
|  | XP\_065056972\_14350-model2.17 | -8.71504 |
|  | XP\_065056972\_14350-model2.18 | -8.67443 |
|  | XP\_065056972\_14350-model2.19 | -8.65193 |
|  | XP\_065056972\_14350-model2.20 | -8.61417 |
|  | XP\_065056972\_14350-model2.21 | -8.43721 |
|  | XP\_065056972\_14350-model2.22 | -8.07892 |
| # cluster 3 - Size: 7 |  |  |
|  | XP\_065056972\_14350-model3 | -10.181 |
|  | XP\_065056972\_14350-model3.1 | -9.63487 |
|  | XP\_065056972\_14350-model3.2 | -9.63487 |
|  | XP\_065056972\_14350-model3.3 | -9.51949 |
|  | XP\_065056972\_14350-model3.4 | -9.26398 |
|  | XP\_065056972\_14350-model3.5 | -9.002 |
|  | XP\_065056972\_14350-model3.6 | -8.80664 |
| # cluster 4 - Size: 12 |  |  |
|  | XP\_065056972\_14350-model4 | -10.0657 |
|  | XP\_065056972\_14350-model4.1 | -9.95439 |
|  | XP\_065056972\_14350-model4.2 | -9.93504 |
|  | XP\_065056972\_14350-model4.3 | -9.65274 |
|  | XP\_065056972\_14350-model4.4 | -9.6046 |
|  | XP\_065056972\_14350-model4.5 | -9.50938 |
|  | XP\_065056972\_14350-model4.6 | -9.42164 |
|  | XP\_065056972\_14350-model4.7 | -9.3302 |
|  | XP\_065056972\_14350-model4.8 | -9.28998 |
|  | XP\_065056972\_14350-model4.9 | -9.26232 |
|  | XP\_065056972\_14350-model4.10 | -9.08122 |
|  | XP\_065056972\_14350-model4.11 | -8.77123 |
| # cluster 5 - Size: 2 |  |  |
|  | XP\_065056972\_14350-model5 | -9.85097 |
|  | XP\_065056972\_14350-model5.1 | -9.45987 |
| # cluster 6 - Size: 4 |  |  |
|  | XP\_065056972\_14350-model6 | -9.84411 |
|  | XP\_065056972\_14350-model6.1 | -9.59266 |
|  | XP\_065056972\_14350-model6.2 | -9.4877 |
|  | XP\_065056972\_14350-model6.3 | -8.41374 |
| # cluster 7 - Size: 1 |  |  |
|  | XP\_065056972\_14350-model7 | -9.74799 |
| # cluster 8 - Size: 1 |  |  |
|  | XP\_065056972\_14350-model8 | -9.70451 |
| # cluster 9 - Size: 4 |  |  |
|  | XP\_065056972\_14350-model9 | -9.68059 |
|  | XP\_065056972\_14350-model9.1 | -9.68059 |
|  | XP\_065056972\_14350-model9.2 | -9.25637 |
|  | XP\_065056972\_14350-model9.3 | -8.62291 |
| # cluster 10 - Size: 1 |  |  |
|  | XP\_065056972\_14350-model10 | -9.60949 |
| # cluster 11 - Size: 10 |  |  |
|  | XP\_065056972\_14350-model11 | -9.55363 |
|  | XP\_065056972\_14350-model11.1 | -9.54089 |
|  | XP\_065056972\_14350-model11.2 | -9.4058 |
|  | XP\_065056972\_14350-model11.3 | -9.40295 |
|  | XP\_065056972\_14350-model11.4 | -9.13408 |
|  | XP\_065056972\_14350-model11.5 | -9.05587 |
|  | XP\_065056972\_14350-model11.6 | -9.02701 |
|  | XP\_065056972\_14350-model11.7 | -8.88084 |
|  | XP\_065056972\_14350-model11.8 | -8.71835 |
|  | XP\_065056972\_14350-model11.9 | -7.1432 |
| # cluster 12 - Size: 5 |  |  |
|  | XP\_065056972\_14350-model12 | -9.5221 |
|  | XP\_065056972\_14350-model12.1 | -9.32161 |
|  | XP\_065056972\_14350-model12.2 | -9.11649 |
|  | XP\_065056972\_14350-model12.3 | -9.01712 |
|  | XP\_065056972\_14350-model12.4 | -8.44734 |
| # cluster 13 - Size: 2 |  |  |
|  | XP\_065056972\_14350-model13 | -9.45791 |
|  | XP\_065056972\_14350-model13.1 | -9.02658 |
| # cluster 14 - Size: 1 |  |  |
|  | XP\_065056972\_14350-model14 | -8.07034 |
| # cluster 15 - Size: 1 |  |  |
|  | XP\_065056972\_14350-model15 | -7.65329 |
| # cluster 16 - Size: 2 |  |  |
|  | XP\_065056972\_14350-model16 | -7.47447 |
|  | XP\_065056972\_14350-model16.1 | -7.38959 |
| # cluster 17 - Size: 1 |  |  |
|  | XP\_065056972\_14350-model17 | -7.279 |
| # cluster 18 - Size: 2 |  |  |
|  | XP\_065056972\_14350-model18 | -7.09995 |
|  | XP\_065056972\_14350-model18.1 | -6.62683 |
| # cluster 19 - Size: 1 |  |  |
|  | XP\_065056972\_14350-model19 | -7.01607 |
| # cluster 20 - Size: 1 |  |  |
|  | XP\_065056972\_14350-model20 | -5.65169 |
| # cluster 21 - Size: 2 |  |  |
|  | XP\_065056972\_14350-model21 | -3.65205 |
|  | XP\_065056972\_14350-model21.1 | -3.24531 |
| # cluster 22 - Size: 2 |  |  |
|  | XP\_065056972\_14350-model22 | -3.18384 |
|  | XP\_065056972\_14350-model22.1 | -3.05631 |
| # cluster 23 - Size: 1 |  |  |
|  | XP\_065056972\_14350-model23 | -3.15091 |
| # cluster 24 - Size: 1 |  |  |
|  | XP\_065056972\_14350-model24 | -3.04997 |
|  |  |  |
